# Acute Kidney Injury and Fever in a Patient After Hematopoietic Stem Cell Transplant

**DOI:** 10.34067/KID.0000000925

**Published:** 2026-01-29

**Authors:** Dayana Abilmona, Yanli Ding, Sabine Karam

**Affiliations:** 1 Department of Internal Medicine, MedStar Health Georgetown University (Baltimore), Baltimore, Maryland; 2 Department of Laboratory Medicine and Pathology, University of Minnesota, Minneapolis, Minnesota; 3 Division of Nephrology and Hypertension, Department of Medicine, University of Minnesota, Minneapolis, Minnesota; 4 Division of Nephrology and Hypertension, Department of Internal Medicine, American University of Beirut, Beirut, Lebanon

**Keywords:** AKI

## Abstract

**Figure:**
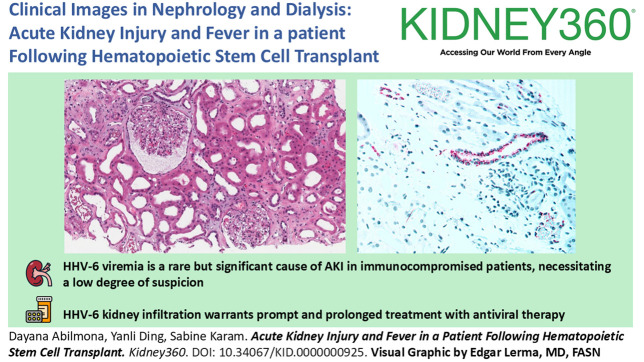

## Case Description

**Figure 1:**
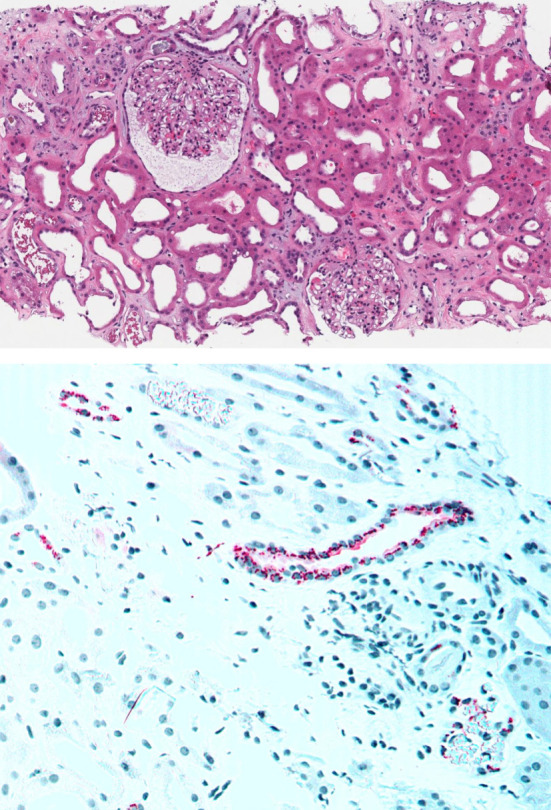
**Acute tubular injury secondary to HHV-6 invasion.** (A) Kidney biopsy showing ATI characterized by focal flattening of epithelium and luminal dilation with mild chronic changes of the parenchyma (hematoxylin and eosin). (B) Immunohistochemistry showing positive cytoplasmic staining in red for HHV-6 in some tubules. ATI, acute tubular injury; HHV-6, human herpes virus 6.

A 73-year-old man with a medical history of coronary artery disease, hypertension, type 2 diabetes, and myeloproliferative neoplasm with secondary JAK2+ myelofibrosis presented with AKI and a creatinine level at 4.25 mg/dl (baseline 0.8–1 mg/dl) in the setting of fever of unclear origin on day 29 posthematopoietic stem cell transplantation. The patient denied any hypotension, any decrease in his urine output, and any intake of nephrotoxic medications such as nonsteroidal anti-inflammatory drugs. His BP was 130/56, and his physical examination was significant for some mucositis only. A urinalysis was positive for proteinuria 2+ and trace hematuria. On further investigation, he was found to have human herpes virus 6 (HHV-6) viremia (103–185 copies/ml), prompting initiation of ganciclovir. He became anuric with a creatinine that peaked at 9.45 mg/dl. A kidney biopsy showed acute tubular injury with mild chronic changes of the parenchyma, including global glomerulosclerosis in about 5% of glomeruli, with mild tubular atrophy and interstitial fibrosis (5%–10% cortex; Figure 1A). Interstitial nephritis or obvious viral cytopathic effects were not seen in the sample. He received five sessions of hemodialysis, and the kidney function started to improve. He completed 2 weeks of ganciclovir, and the HHV-6 viral load decreased to 1406 copies/ml. However, his kidney function started declining again, warranting hemodialysis. The number of HHV-6 copies was checked again and found to be at 20,081 copies/ml 2 days after stopping ganciclovir. Viral therapy was reinitiated, with subsequent AKI resolution. Kidney biopsy tissue was thereafter sent for viral staining, and positive cytoplasmic staining for HHV-6 antigen was seen in a few tubules (Figure 1B).

## Discussion

HHV-6 is a lymphotropic herpesvirus of emerging clinical significance in the immunocompromised host.^1^ The presence of HHV-6 in renal tissue has been mostly reported in kidney transplant patients and has been correlated with transplant rejection.^2^ Kidney infection with HHV-6 has also been reported in a case of drug-induced hypersensitivity syndrome/drug rash with eosinophilia and systemic symptoms where the biopsy showed granulomatous tubulointerstitial nephritis with HHV-6 proliferation in tubular epithelial cells.^3^ Our case is to our knowledge the first of isolated acute tubular injury due to HHV-6 invasion in a patient with hematopoietic stem cell transplantation, severe enough to warrant hemodialysis. Our patient improved on ganciclovir therapy with complete recovery of his kidney function. HHV-6 is a rare but significant cause of severe AKI, necessitating a low degree of suspicion in immunocompromised patients and likely a prolonged course for the best outcome.

## Teaching Points

- HHV-6 viremia is a rare but significant cause of AKI in immunocompromised patients, necessitating a low degree of suspicion.
- HHV-6 kidney infiltration warrants prompt and prolonged treatment with antiviral therapy.
